# Successful treatment of an infant with congenital bile acid synthesis disorder type 3 by ursodeoxycholic acid: a case report

**DOI:** 10.1186/s13256-022-03365-z

**Published:** 2022-04-07

**Authors:** Weiqian Mo, Feng Wang, Chuanen Zhou, Tinghe Ma, Zhaojun Pan, Min Xie, Haoyan Ren, Yongwu Xie

**Affiliations:** Department of Pediatrics, Zhuhai Women and Children’s Hospital, 543 Ningxi Road, Xiangzhou District, Zhuhai, Guangdong China

**Keywords:** Congenital bile acid synthesis disorder, *CYP7B1* gene, Oxysterol 7α-hydroxylase, Ursodeoxycholic acid

## Abstract

**Background:**

Congenital bile acid synthesis disorder type 3 caused by oxysterol 7α-hydroxylase deficiency is an extremely rare genetic liver disease. As it may cause rapid progression to end-stage liver disease, a high cautiousness in diagnosis and early treatment are required. Here we describe the first case of congenital bile acid synthesis disorder type 3 in China that was confirmed by genetic analysis.

**Case presentation:**

A 5-month-old Chinese male infant suffered skin yellowing since birth. The patient showed significantly increased alanine transaminase, aspartate transaminase, and total and direct bilirubin levels, and enlarged liver at admission. Whole-exome sequencing confirmed homozygous mutation in the *CYB7B1* gene that encodes oxysterol 7α-hydroxylase. Ursodeoxycholic acid treatment significantly mitigated the condition of the patient and lowered biochemical indicators. Unfortunately, the patient developed septicemia and gave up treatment.

**Conclusions:**

The patient was successfully treated with ursodeoxycholic acid, which has not been reported previously. Ursodeoxycholic acid replacement therapy is an effective and affordable treatment for congenital bile acid synthesis disorder type 3 caused by oxysterol 7α-hydroxylase deficiency.

## Background

Congenital bile acid synthesis disorders are rare genetic diseases caused by enzyme defects of bile acid synthesis process, accounting for about 1–2% of cholestatic diseases in children, most of which belong to autosomal recessive inherited diseases and involve at least 17 enzymes [1]. Congenital bile acid synthesis disorder type 3 (CBAS3) is an extremely rare subtype caused by deficiency of oxysterol 7α-hydroxylase encoded by the cytochrome P450 7B1 (*CYP7B1*) gene [2], manifesting obvious cholestasis in the neonatal period with progressive aggravation and liver enlargement. This rare inborn error of bile acid synthesis responds poorly to bile acid therapy because the progression to cirrhosis is rapid and occurs at an early age. Steroids are rarely used because they can worsen the prognosis of infants with CBAS3 [3, 4]. So far, only five patients with CBAS3, four male and one female, have been reported [5–9]. Two patients died in infancy either before or after liver transplantation [5, 7], two patients survived after allograft liver transplantation from parental donors [6, 9], and one patient showed normal liver function after about 5 years of treatment with chenodeoxycholic acid (CDCA), which is the only case treated successfully with drug [8]. In the same case report, the authors stated that ursodeoxycholic acid (UDCA) treatment worsened the patient’s condition.

Here we report a case of CBAS3 caused by mutation of the *CYP7B1* gene, which was confirmed by genetic testing. The patient was treated with UDCA and demonstrated a significant improvement both in laboratory tests and in symptoms, suggesting that UDCA may be an effective and economical treatment method for congenital bile acid synthesis disorders.

## Case presentation

The patient (a Chinese male, 5 months old) was admitted to our department on 17 May 2020 due to skin yellowing for more than 4 months, worsened for 1 month, and fever for 1 day. The patient was the first pregnancy and first delivered child of the mother and was born at full term with birth weight of 2850 g. The parents were healthy, denied history of intermarriage and genetic metabolic diseases, and denied family history of hepatobiliary diseases. The patient developed skin yellowing 3 days after birth, and the yellowing was not alleviated after taking Chinese medicine intermittently (the specific medicine was unknown). Since the onset of skin yellowing, the patient was in good spirits but had poor appetite with yellow stool and urine. Physical examination showed body weight 6 kg, clear mind, good spirits without special facial appearance, developmental delay, unstable head up, moderate yellow staining of the skin and sclera, no abnormalities on heart and lung auscultation, soft abdomen, and redness and swelling around the umbilicus. The liver was 3 cm below the right rib ridge, with medium texture and clear boundary, and the spleen was unreached.

**Fig. 1:**
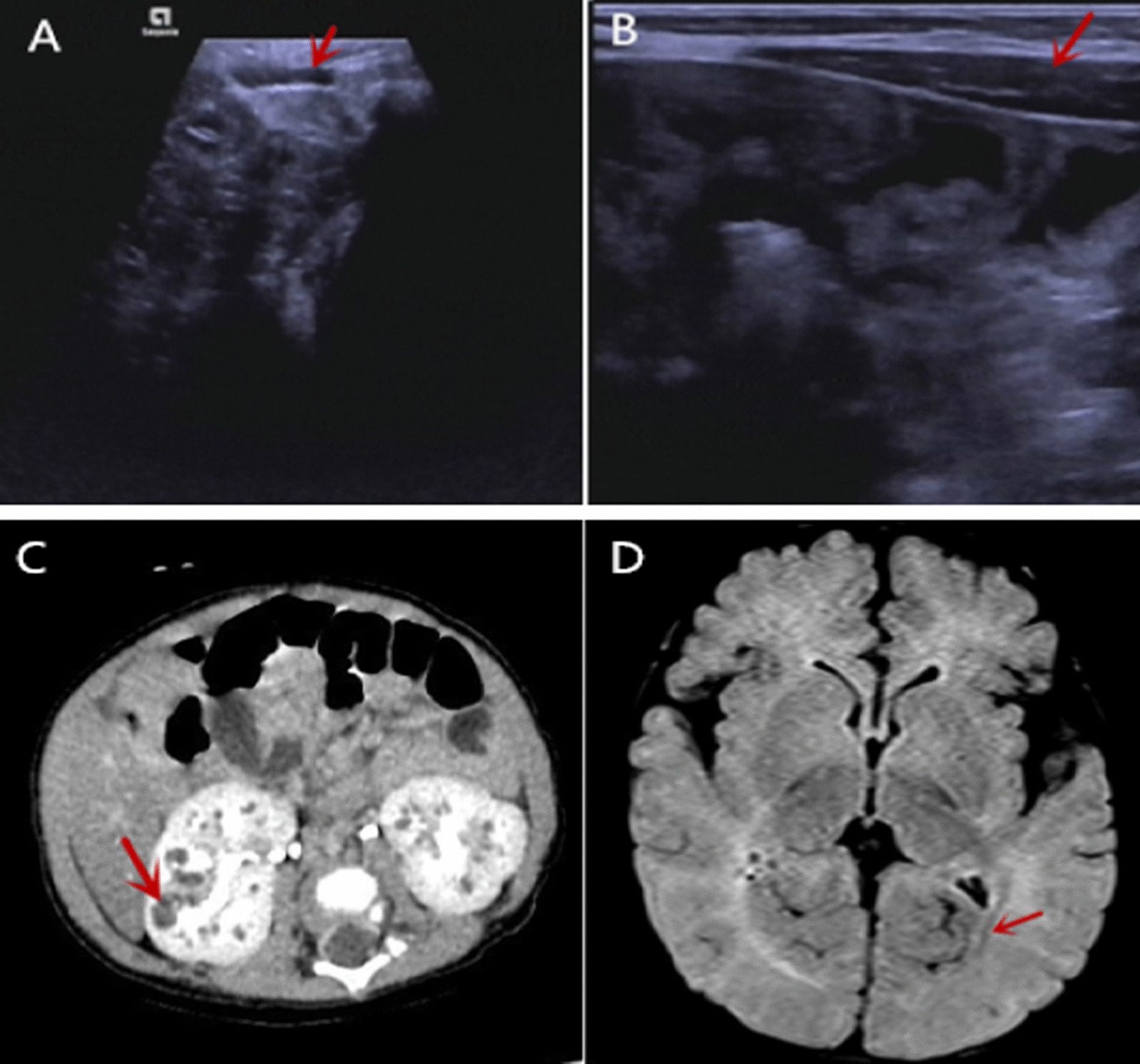
Umbilical sinus, polycystic kidney, and myelination dysplasia of the patient. **A**, **B** Umbilical color Doppler ultrasound showed abnormal echo (red arrow) in the umbilical area. **C** Abdominal CT showed that the volume of both kidneys was increased, and multiple small sac-like unenhanced areas of different sizes were detected (red arrow) in the renal medulla. **D** Cranial MRI showed the left cisterna and the anterior temporal space were slightly widened, and the posterior horn of bilateral ventricles manifested small patchy abnormal signal foci (red arrow)

Abdominal ultrasound showed that the intrahepatic bile duct was well revealed and the biliary tract was unobstructed. Umbilical color Doppler ultrasound showed abnormal echo in the umbilical area, suggesting umbilical sinus (Fig. 1A, B). Abdominal enhanced CT scan showed that liver was slightly enlarged and the density of parenchyma was diffusely reduced; the volume of both kidneys was increased, parenchymal density was not uniform, and multiple small sac-like unenhanced areas of different sizes were detected, mainly in the renal medulla, suggesting infant polycystic kidneys (Fig. 1C). Cranial MRI showed multiple punctate abnormal signals in the bilateral cerebellar hemispheres and near the posterior horn of the right ventricle, suggesting the possibility of multiple small hemorrhage lesions; the bilateral prefrontal space, the left cisterna, and the anterior temporal space were slightly widened, and the posterior horn of bilateral ventricles manifested small patchy abnormal signal foci, suggesting the possibility of myelination dysplasia (Fig. 1D).

**Fig. 2:**
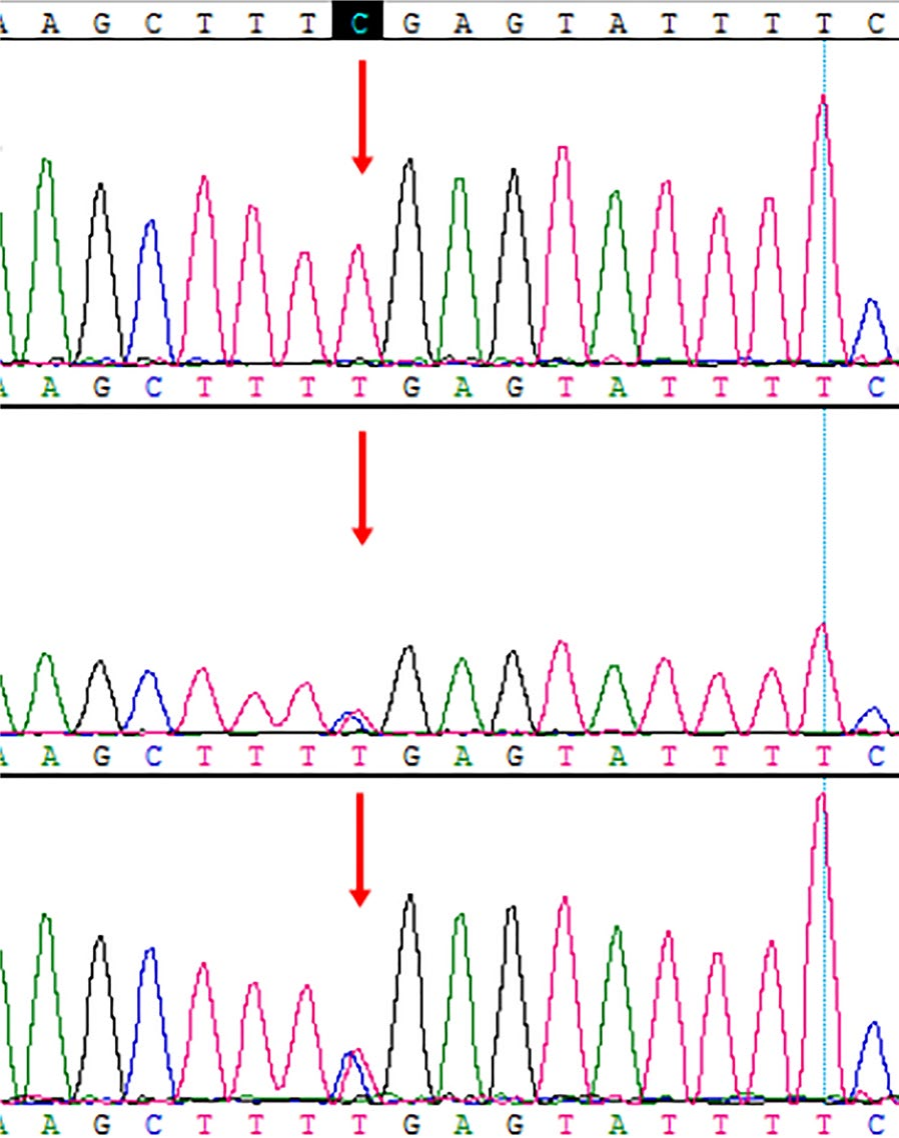
Genetic analysis sequencing results. The patient showed a homozygous C>T mutation (red arrow, top panel), and both parents showed heterozygous mutation at the same locus (red arrow, middle and lower panel).

Whole-exome sequencing test was performed in the patient and the parents. The patient showed homozygous mutation in the *CYP7B1* gene NM_004820.3:c.334C>T(p.Arg112*). The pathogenicity of this mutation has been reported [2]. According to the ACMG guidelines, this mutation is a pathogenic variant. Both parents of the child showed heterozygous mutation at this locus with normal phenotype, conforming to the rule of autosomal recessive inheritance. Figure 2 shows the sequencing results of the patient, father, and mother, from top to bottom panel, respectively.

After admission, the patient was given symptomatic treatments, including anti-infection, liver protection, and choleretic treatment. The yellow staining of the skin was not significantly improved. The patient was given oral UDCA 50 mg three time a day from 21 May to 12 June 2020, 100 mg twice a day from 13 to 18 June, and 125 mg twice a day from 19 June to 1 August. After symptoms improved, the patient was discharged from hospital on 21 June 2020. The patient was regularly followed up for biochemical examinations and check-ups. The parents stopped the drug without doctor’s permission since 2 August, and resumed drug administration at 100 mg twice a day since 19 October 2020 when biochemical examination showed that liver function had deteriorated. On 21 January 2021, the patient was admitted to our department again due to unconsciousness. Unfortunately, septicemia occurred on 21 January 2021, and the family decided to give up treatment on 23 January 2021 and left the hospital.

**Fig. 3:**
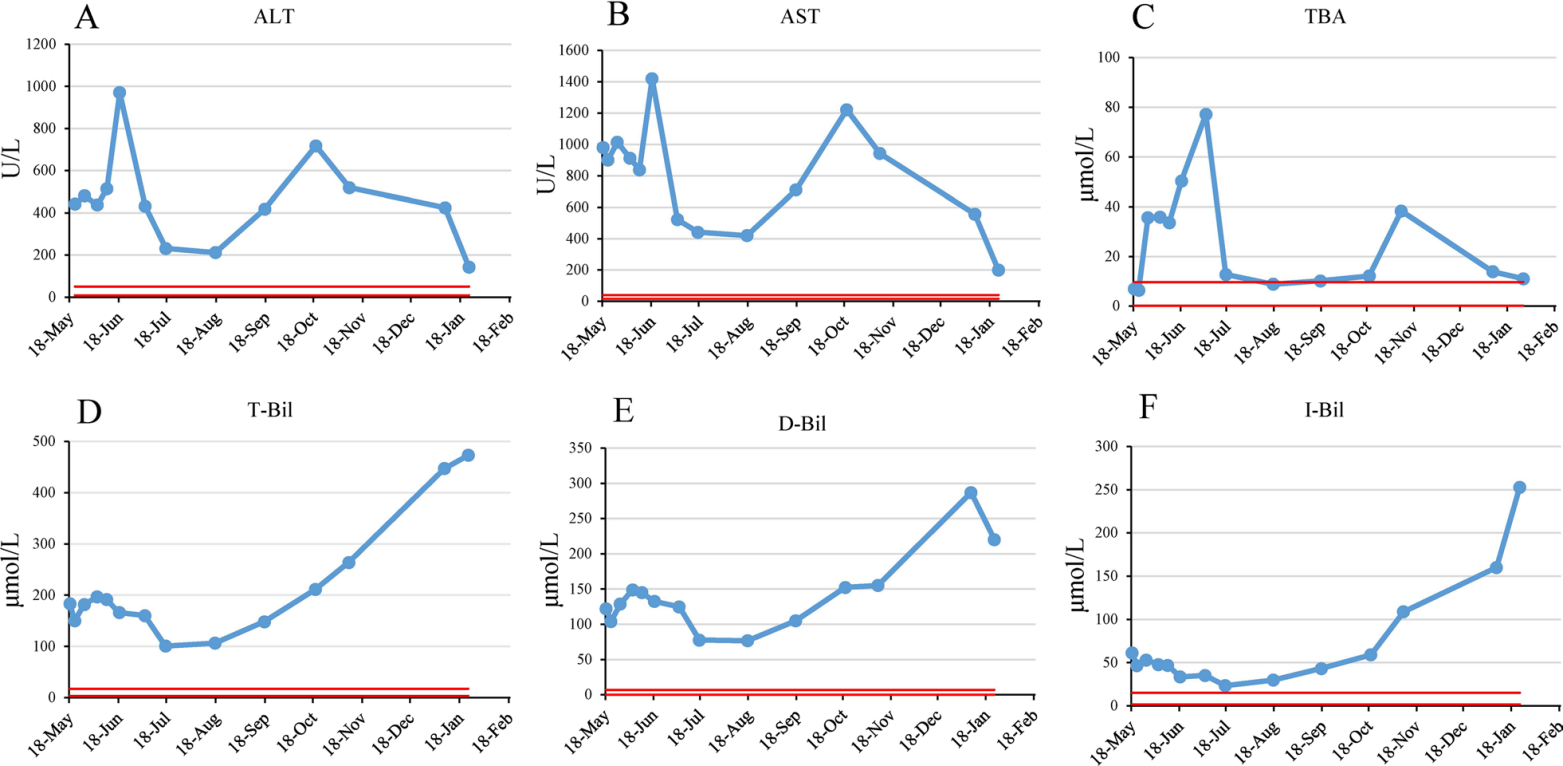
Liver function and bilirubin level. **A**–**F** Blood biochemical examination results from May 2020 to January 2021. **A** ALT; **B** AST; **C** total bile acid; **D** total bilirubin; **E** direct bilirubin; **F** indirect bilirubin. Red lines represent the normal range of each indicator

Blood biochemical examination results of the patient are summarized in Tables 1 and 2 and Fig. 3, revealing that the levels of most indicators increased shortly after UDCA administration, probably because liver function improvement takes much longer after the drug has its pharmacological effects. With the use of UDCA, the symptoms of the patient improved and all indicators demonstrated a declining trend until 2 August, when the patient stopped taking the medicine without consulting doctor. Thereafter, all indicators increased significantly and symptoms deteriorated until the patient resumed the drug again on 19 October 2020, since when some of the indicators declined again. The last two blood tests, performed on 8 and 23 January 2021, respectively, showed significant enzyme-bilirubin dissociation. Combined with the fact that the patient developed septicemia on 21 January 2021, this suggests that significant hepatocyte necrosis might have occurred since early January 2021 and might be part of the cause of the occurrence of septicemia. However, because the parents refused liver biopsy, the exact explanation for the test results and cause of septicemia is not available. No obvious abnormal products were detected in urine mass spectrometry analysis.

**Table 1:** Liver function and bilirubin level

Date	ALT (U/L)	AST (U/L)	GGT (U/L)	TBA (μmol/L)	T-BIL (μmol/L)	D-BIL (μmol/L)	I-BIL (μmol/L)
5/18/20	443.1	979.6	23.9	6.9	182.9	121.7	61.2
5/21/20	441.1	899.1	24.6	6.3	150.1	103.8	46.3
5/27/20	479.9	1013.7	31.9	35.6	181.5	128.6	52.9
6/04/20	437.0	912	35	35.8	196.4	148.7	47.7
6/10/20	513.8	837.5	30.3	33.5	191.3	144.7	46.6
6/18/20	970	1420	42	50.3	165.8	132.3	33.5
7/04/20	431	521	30	77.1	159.9	124.7	35.2
7/17/20	231	440	22	12.7	100.7	77.4	23.3
8/17/20	211	419	28	8.8	106.4	76.6	29.8
9/17/20	418	709	28	10.1	147.9	104.8	43.1
10/19/20	717	1221	31	12.1	211.2	152.2	59
11/09/20	518.8	942	28	38.3	263.7	154.9	108.8
01/08/21	424	555.6	35	13.8	446.8	286.8	160
01/23/21	142.3	198.8	20	11	472.8	220	252.8

**Table 2:** Coagulation function

Date	PT (seconds)	PT-INR	FIB (g/L)	APTT (seconds)	TT (seconds)	AT-III (%)	FDP (μg/mL)	D-dimer (mg/L)
05/17/20	20.0	1.76	2.66	35.3	18	33	5.9	2.9
05/18/20	17.7	1.55	2.74	33.6	18.4			
05/19/20	18.0	1.58	2.42	35.5	18.6	32	5.2	2.4
05/21/20	16.8	1.47	2.27	33.7	19.4	34	4.3	2.34
05/23/20	17.8	1.56	2.01	34.2	19.5			
05/27/20	17.4	1.52	2.21	32.1	20.1			
06/04/20	15.7	1.37	1.36	27.6	22.5			
06/10/20	17.9	1.57	2.36	37.9	20.2	35	4.3	2.01
06/18/20	16.8	1.47	1.8	36.9	22			
07/04/20	18.8	1.65	2.63	37.6	19.2	33	2.5	0.62
07/17/20	17.9	1.57	2.39	35.6	19.2	32	2.5	1.02
08/17/20	16	1.4	2.29	27.9	18.9	47	2.8	0.76
09/17/20	19.4	1.71	3.23	32.5	18.7	42	2.8	0.86
10/19/20	22.4	1.98	2.35	35.6	20.9	23	3.1	0.94
11/09/20	22.6	1.99	2.26	35.6	19.9	38	2.8	0.75
01/08/21	33.8	3.05	1.39	53.1	23.5	19	15.8	7.85
01/23/21	43.4	3.98	1.29	62.8	23.1	5	14.1	5.86

## Discussion and conclusion

Congenital bile acid synthesis disorders are extremely rare genetic metabolic disorders caused by enzyme defects and involving the pathways for bile acid synthesis from cholesterol in the liver [1]. We describe the first case of congenital bile acid synthesis disorder type 3 caused by oxysterol 7α-hydroxylase deficiency in China. The clinical manifestations and prognosis of this disease are not clearly defined due to the rareness of this disease. The age of these patients at onset is mostly 1–20 weeks after birth. All patients manifested jaundice, enlarged liver, significantly elevated ALT and AST, normal GGT, and normal or elevated total bile acid. Other clinical manifestations include coagulopathy, hypoglycemia, steatorrhea, malabsorption of lipid-soluble vitamins, rickets, and growth retardation.

The patient in this case showed obvious cholestasis in the neonatal period, accompanied by skin itching, and umbilical area redness with frequent secretions. Ultrasound examination revealed umbilical sinus and infant polycystic kidney changes. Moreover, the child also demonstrated physical and nervous system developmental delays. Head MRI revealed multiple small hemorrhage lesions, slightly widened bilateral prefrontal space, left cisterna, and pretemporal space, and myelination dysplasia. Severe infections occurred during the course of the disease, which may be related to the deficiency of the body’s immune function due to liver dysfunction. The umbilical sinus of this patient has not been reported in other cases, and whole-exome sequencing did not detect other related pathogenic gene mutations. Therefore, whether the umbilical change is associated with the *CYP7B1* gene awaits further study. This patient had polycystic kidneys, the first reported case of oxysterol 7-α-hydroxylase deficiency also had renal cyst changes, and certain types of congenital bile acid synthesis disorders have also been reported with renal cyst changes [10, 11]. The mechanism of renal cyst formation is not clear but may be related to the abnormal bile acid metabolites produced in bile acid metabolism disorders [7, 12]. The physical retardation of this child is considered to be caused by protein and lipid metabolism disorders and insufficient energy intake due to long-term liver function insufficiency and cholestasis. The developmental delay of the nervous system and MRI imaging changes of the patient may be related to the involvement of oxysterol 7α-dehydrogenase in extrahepatic tissues, such as the degradation of cholesterol in the brain and the metabolism of neurosteroids [9].

Blood biochemical examination of patients with CBAS3 may reveal significant hyperbilirubinemia, with elevation of mainly direct bilirubin, and significantly elevated serum transaminases, but normal level of GGT and normal or slightly increased total bile acid. PT and aPTT are significantly prolonged, which may be caused by vitamin K malabsorption and liver insufficiency. Liver biopsy shows cholestasis, obvious hepatic giant cell transformation, extensive fibrosis, bile duct arrangement disorder, and small bile duct hyperplasia [13]. The liver function test results of this patient were consistent with the liver function changes of congenital bile acid synthesis disorder, and the PT and aPTT were prolonged. The patient did not undergo liver biopsy, thus pathological changes of the patient’s liver were unavailable.

The current diagnostic methods for CBAS3 mainly include genetic testing and urine mass spectrometry. The *CYP7B1* gene mutation was initially discovered in hereditary spastic paraplegia type 5 [14, 15]. The *CYP7B1* gene is located on chromosome 8q21.3, with full length of 202.66 kb, containing six exons and encoding cytochrome P-450 and oxysterol 7α-hydroxylase. In this case, a homozygous mutation (c.334C>T) was identified in the third exon of the *CYP7B1* gene. Moreover, this mutation is a known pathogenic mutation. Multiple urine mass spectrometry examinations were performed in this case, while no special metabolites or monohydroxy bile acids (3β-hydroxy-5-cholanoic acid and 3β-hydroxy-5-cholenoic acid) were detected. We postulated that these test results might be affected by urine concentration and medication to certain extent. Genetic testing, which requires only 2–3 mL of peripheral blood and usually achieves stable results, is not affected by disease status or medication. Although genetic testing has the disadvantage of high cost, it is a stable and reliable method for diagnosis of this disease.

Among the very few reported CBAS3 cases, successful drug treatment was reported in only one patient who took chenodeoxycholic acid before liver function deteriorated significantly, and achieved symptom improvement and restoration of liver functions [8]. In this case, the patient was treated with ursodeoxycholic acid. Liver function and bilirubin indicators initially increased after drug treatment. The possible explanation is that blood biochemical changes are the final manifestation of a series of changes induced by the drug. This initial increase is also consistent with the findings reported by Dai *et al.* [8] that, after UDCA treatment, their patient’s condition worsened. However, after prolonged administration of UDCA, both the physical condition and blood biochemical indicators of our patient improved. Unfortunately, the parents of the patient stopped using the drug without consulting us, resulting in a worsening of the symptoms and biochemical examination results. After several attempts, we persuaded the parents to resume the treatment, thus the patient’s symptoms were improved and blood indicator levels decreased again. Unfortunately, the patient developed septicemia on 21 January 2021, and the family gave up treatment. Based on our communication with the patient’s family, we suspect that the family may not strictly follow our prescription, which may partially lead to the deterioration of the patient’s condition.

Although liver transplantation may be the cure for this disease, due to the unavailability of donors and high cost of the surgery, available effective drug treatment will definitely benefit more patients with this type of disease. Here we report an effective and economical treatment for CBAS3, ursodeoxycholic acid replacement therapy. However, due to the rareness of this disease, treatment efficacy and prognosis await further observation.

## Data Availability

Not applicable.

## Abbreviations

CBAS3: Congenital bile acid synthesis disorder type 3
CYP7B1: Cytochrome P450 7B1
UDCA: Ursodeoxycholic acid
ALT: Alanine transaminase
AST: Aspartate transaminase
GGT: γ-Glutamyl transferase
aPTT: Activated partial thromboplastin time
PT: Prothrombin time
